# Optimization of Thick-Walled Viscoelastic Hollow Polymer Cylinders by Artificial Heterogeneity Creation: Theoretical Aspects

**DOI:** 10.3390/polym13152408

**Published:** 2021-07-22

**Authors:** Anton Chepurnenko, Stepan Litvinov, Besarion Meskhi, Alexey Beskopylny

**Affiliations:** 1 Strength of Materials Department, Faculty of Civil and Industrial Engineering, Don State Technical University, Rostov-on-Don 344000, Russia; litvstep@gmail.com; 2 Department of Life Safety and Environmental Protection, Faculty of Life Safety and Environmental Engineering, Don State Technical University, Rostov-on-Don 344000, Russia; reception@donstu.ru; 3 Department of Transport Systems, Faculty of Roads and Transport Systems, Don State Technical University, Rostov-on-Don 344000, Russia

**Keywords:** polymers, heterogeneity, creep, stress–strain state, equal-strength structures, equally stressed structures, thick-walled shells, optimization, high-density polyethylene, hydroxyapatite, centrifugation

## Abstract

A theoretical solution of the problem of thick-walled shell optimization by varying the mechanical characteristics of the material over the thickness of the structure is proposed, taking into account its rheological properties. The optimization technique is considered by the example of a cylindrical shell made of high-density polyethylene with hydroxyapatite subjected to internal pressure. Radial heterogeneity can be created by centrifugation during the curing of the polymer mixed with the additive. The nonlinear Maxwell–Gurevich equation is used as the law describing polymer creep. The relationship of the change in the additive content along with the radius r, at which the structure is equally stressed following the four classical criteria of fracture, is determined in an elastic formulation. Moreover, it is shown that a cylinder with equal stress at the beginning of the creep process ceases to be equally stressed during creep. Finally, an algorithm for defining the relationship of the additive mass content on coordinate r, at which the structure is equally stressed at the end of the creep process, is proposed. The developed algorithm, implemented in the MATLAB software, allows modeling both equally stressed and equally strength structures.

## 1. Introduction

Thick-walled cylindrical shells are widely used in the gas, oil refining, chemical, petrochemical, and food industries, in the form of pipes, tanks, high-pressure vessels, and others. From the solution of the Lamé problem, it is known that for a homogeneous thick-walled cylinder under internal pressure, the maximum circumferential stresses are at the inner surface. Thus, in this case, the strength of the material is not fully implemented in these types of structures.

When creating an artificial inhomogeneity of the material, the stress–strain state in thick-walled cylindrical shells subjected to internal pressure can change significantly. The analysis of the stress–strain state of radially inhomogeneous thick-walled cylinders with different laws of variation in the modulus of elasticity along the radius, including exponential, power-law, etc., was carried out in [1,2,3,4,5]. This analysis showed that in contrast to homogeneous structures, maximum stresses do not necessarily occur at the inner surface of the shell.

For optimal use of the material strength, it is necessary to ensure that the limiting state occurs simultaneously at all points, that is, to create an equal-strength structure. For example, if the elastic modulus is reduced at the points of a thick-walled cylinder with higher stresses, then the stresses in them decrease, and vice versa [6,7,8]. Thus, when changing the modulus of elasticity of the material in the structure’s thickness according to a specific law, it is possible to achieve the constant equivalent stress according to any failure criterion. In this case, the structure is equally stressed. An equally stressed structure can be of equal strength if the strength of the material does not change when the elastic modulus changes.

The described idea is based on the inverse method of structure optimization. The essence of the approach is to find such laws of variation in material characteristics, in which the stress–strain state of the structure is given [9]. In [10], a technique for achieving constant hoop stress throughout the thickness of a cylinder subjected to hydrostatic boundary loads is proposed. In [9,11,12], solutions are presented for the problem of finding the law of change in the modulus of elasticity of a material, in which thick-walled cylinders and spheres subjected to the action of internal pressure are equally stressed according to the criterion of maximum shear stresses and the maximum elastic distortional energy criterion. In [13], the solution to this problem is presented based on Mohr’s failure criterion. It is shown that from the solution based on Mohr’s theory, it is possible to obtain, as special cases, solutions based on three classical failure criteria: the criterion of maximum normal stresses, the criterion of maximum deformations, and the criterion of maximum shear stresses. The works [14,15] consider the model of an equally stressed cylinder based on the Balandin failure criterion.

In [16], the plane strain problem for a functionally graded cylinder subjected to both normal and tangential nonuniform external pressure is solved. Both the power and exponential laws of the shear modulus were considered. In addition, the authors managed to identify a radial variation pattern in which the linear combination of the radial and the hoop stress can follow a given distribution.

In articles [17,18], in addition to concentrated loads, temperature effects are taken into account when solving optimization problems. In [19,20], the technique of varying the material’s mechanical characteristics is considered to create equal-strength bar structures.

The practical implementation of an equally stressed cylinder manufacture can be performed according to the method proposed in [21]. First, the polymer mass is mixed with a finely dispersed mineral filler. Then the composite is placed into a cylindrical shape that rotates as the polymer cures. In this case, the solid phase is displaced to the periphery under the action of inertial forces, nonuniformly distributed along the cylinder radius. As a result, the modulus of elasticity is changed. By changing the type of filler, its percentage, and the speed of rotation of the centrifuge, it is possible to bring the function of variating the modulus of elasticity closer to the required one. This method is widely used in the production of centrifuged concrete [22,23,24,25].

The mechanical properties of some polymers can also be modified by exposing them to light of different intensities [26]. For example, for a fiber-reinforced composite, the volume fraction of the fibers and their orientation in the direction of thickness can vary to obtain a suitable modulus gradation [27].

In all the works above, the solution of optimization problems is performed in a linear setting. There are few publications on the analysis of heterogeneous thick-walled shells taking into account nonlinearity. In [28], the analysis of dilatation deformations of a functionally graded material (FGM) second-order elastic thick-walled spherical shell is carried out. The material is assumed to be isotropic and incompressible. In [29], a closed-form solution for a hollow multilayer sphere made of transversally isotropic and hyper elastic FGM is obtained. The axisymmetric problem for a nonlinear elastic hollow sphere is also considered in [30]. In [31], the same methods of a similar problem for a thick-walled cylinder are used. In [32], a nonlinear finite element analysis of thermo-elasticity of a thick-walled FGM cylinder is carried out, taking into account the dependence of material properties on temperature. In [33], the analysis of thermal loads of a thick-walled cylinder is carried out, taking into account nonlinear kinematic hardening. The load is represented by constant internal pressure and cyclic temperature gradient loading.

An essential aspect in the calculations of radially inhomogeneous cylinders is the experimental verification of deformation models. The paper [34] presents experimental tests of hollow bamboo cylinders for the action of internal pressure. Bamboo is a natural material with radial inhomogeneity. The results presented in [34] confirm the reliability of the theoretical solutions considered above. Additionally, in [35], experimental studies of radially inhomogeneous cylinders made of epoxy resin with a diabase flour filler were carried out, which showed a good agreement between the experiment and theory.

Many materials are characterized by the phenomenon of creep, which can significantly affect the stress–strain state. However, there are relatively few works in the literature on the analysis of the creep of inhomogeneous structures in the form of thick-walled cylinders and spheres [36,37], and optimization problems with the creep taken into account have not been considered previously. Therefore, the aim of this work is to solve the problem of optimizing a thick-walled cylinder taking into account the material creep.

## 2. Materials and Methods

**Figure 1:**
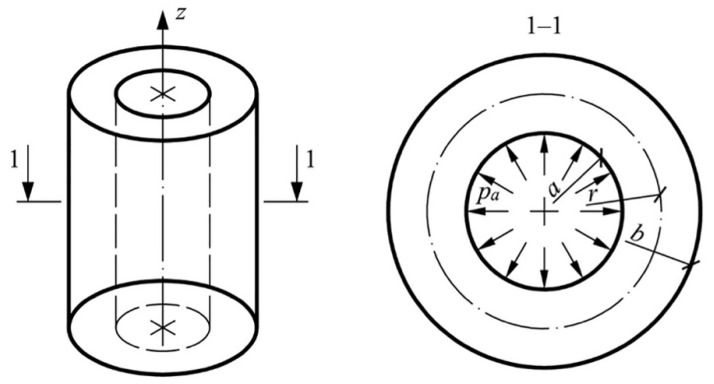
Calculation scheme.

The optimization algorithm using the example of a thick-walled cylinder made of high-density polyethylene (HDPE) with the addition of hydroxyapatite is considered below. A cylinder with an inner radius *a* and an outer radius *b* under the action of an internal pressure *p_a_* is in the condition of plane strain (Figure 1).

For many types of polymers, the generalized Maxwell–Gurevich equation shows good agreement with the experimental data [38], which in the case of a triaxial stress state has the form:

(1) $$\begin{array}{c} \frac{\partial \varepsilon_{ij}^{*}}{\partial t}=\frac{f_{ij}^{*}}{\eta^{*}},\;i=r,\theta ,z;j=r,\theta ,z;\\ f_{ij}^{*}=\frac{3}{2}\left(\sigma_{ij}-p\delta_{ij}\right)-E_{\infty }\varepsilon_{ij}^{*};\\ \eta^{*}=\eta_{0}^{*}\;\exp\left(-\frac{\left|f_{max}^{*}\;\right|}{m^{*}}\;\right);\;f_{max}^{*}={\left|\frac{3}{2}\left(\sigma_{rr}-p\;\right)-E_{\infty }\varepsilon_{rr}^{*}\right|}_{max} \end{array}$$

where $\varepsilon_{ij}^{*}$ is the creep strain, *E*_∞_—high elasticity deformations modulus, $\eta_{0}^{*}$—initial relaxation viscosity, $\delta_{ij}$—Kronecker symbol, $p=\left(\sigma_{r}+\sigma_{\theta }+\sigma_{z}\right)/3$—the average stress, $m^{*}$—velocity modulus, and index *rr* corresponds to the directions of principal stresses.

A detailed study of a hydroxyapatite additive (HA) effect on HDPE properties was presented in [39]. In [38], the creep curves of the modified HDPE are processed to obtain the dependence of the physical and mechanical parameters of the material on the various percentages of HA additives:

(2) $$\begin{array}{c} E\left(HA\right)=694+1251\cdot HA\;\left[\mathrm{MPa}\right];\\ E_{\infty }\left(HA\right)=228.9+1093\cdot HA\;\left[\mathrm{MPa}\right] \end{array}$$

where *HA* is the hydroxyapatite (wt. %).

Thus, when 30% hydroxyapatite is added into high-density polyethylene, the elastic modulus can increase up to 1.5 times.

The optimization algorithm in the elastic setting is as follows:

- At the first stage, a homogeneous structure is calculated numerically, by the finite difference method or by the finite element method, at *E* = const, and equivalent stresses are determined according to a given strength theory. Using the finite-difference method to determine the stress–strain state of the cylinder, Equation (3) [38] can be used:
  (3) $$\sigma_{r}^{\prime \prime }+\varphi \left(r\right)\sigma_{r}^{\prime }+\psi \left(r\right)\sigma_{r}=0$$
  where $\varphi \left(r\right)=\frac{3}{r}-\frac{E^{\prime }}{E};\psi \left(r\right)=-\frac{1}{r}\left[\frac{1-2\nu }{1-\nu }\frac{E^{\prime }}{E}\;\right].$

The dash here denotes the derivative with respect to *r*. When *E* = const, *E*’ is equal to zero. The boundary conditions are:

(4) $$\sigma_{r}\left(a\right)=-p_{a};\;\;\sigma_{r}\left(b\right)=0$$

Stresses $\sigma_{\theta }$ can be defined as

(5) $$\sigma_{\theta }\left(r\right)=r\sigma_{r}^{\prime }+\sigma_{r}$$

- The modulus of elasticity is corrected at each node by the formula:
  (6) $$E_{i}:=E_{i}\frac{1+\sigma_{eqv,i}/\sigma_{0}}{2}$$
  where $\sigma_{eqv,i}$—equivalent stress at the *i*-th node and *σ*_0_ is the equivalent stress on the inner surface at *r* = *a*.

In this case, the elastic modulus at the inner surface remains constant.

- The calculation is performed with the corrected values of the modulus of elasticity using Equation (3), or the finite element method, and the equivalent stresses are also determined.

Steps 2–3 are repeated until the difference between the elastic modulus values at the outer surface at the previous, and the next steps become less than a predetermined error.

Taking the creep into account, minor adjustments are made to the optimization algorithm, which is discussed below.

## 3. Results

### 3.1. Optimization Results in Linear Elastic Setting

The calculation with the following initial data is performed: *a* = 15 cm, *b* = 22 cm, ν = 0.3, *p*_a_ = 1 MPa. The initial value of the elastic modulus of HDPE without additives is *E*_0_ = 694 MPa.

**Figure 2:**
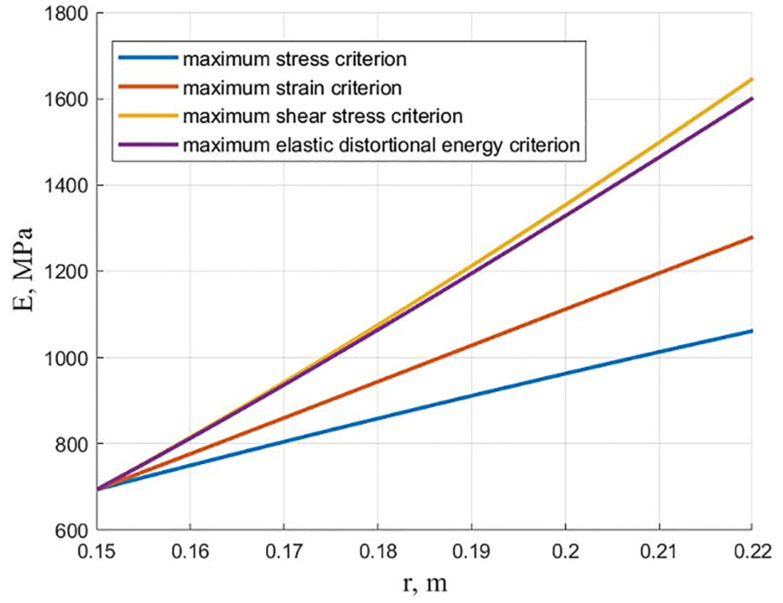
The dependencies of the modulus of elasticity on the radius for an equally stressed cylinder using various failure criteria.

Figure 2 shows the dependencies of the modulus of elasticity on the radius for an equally stressed cylinder at the initial moment. Four classical failure criteria were used: maximum stress criterion, maximum strain criterion, the Tresca criterion of maximum shear stress, and the von Mises criterion of maximum elastic distortional energy.

A comparison was made with the analytical solutions presented in [9,11,12,40] for all the curves obtained. The discrepancy between the results is insignificant. It can be seen from the presented graphs that the most significant difference between the elastic moduli on the inner and outer surfaces is obtained according to the maximum shear stress criterion, and the smallest is according to the maximum stress criterion. Thus, the criteria of maximum shear stress and maximum elastic distortional energy give relatively close results.

If the dependence of the elasticity modulus on the hydroxyapatite content is known, the content of hydroxyapatite can be found by the formula:

(7) $$HA,\;\%=\frac{E-694\;\mathrm{MPa}}{1251\;\mathrm{MPa}}\cdot 100\%$$

**Figure 3:**
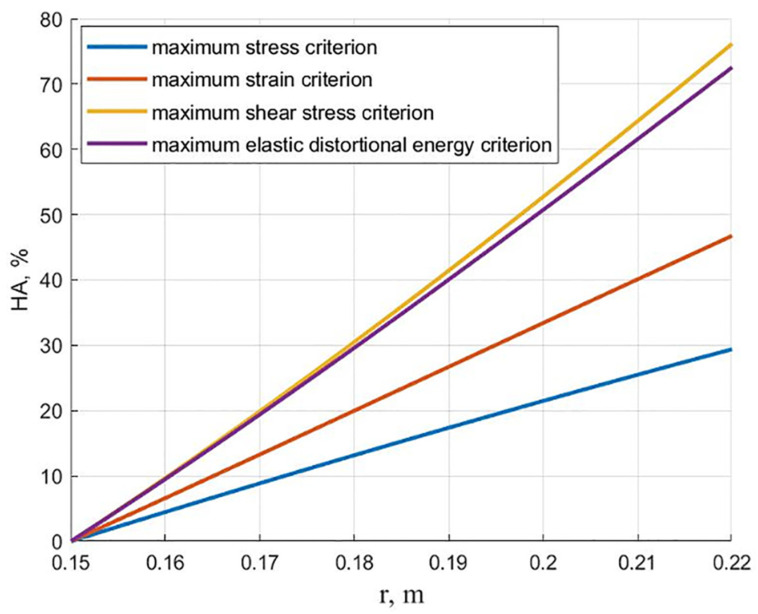
The relationship of the hydroxyapatite content on the radius for equally stressed cylinders according to various failure criteria.

Figure 3 shows the dependencies of the hydroxyapatite content on the radius for an equally stressed cylinder, corresponding to four failure criteria. It can be seen from the presented graphs that, except for the maximum stress criterion, in other cases, the content of hydroxyapatite is beyond the limits of experimental data [9,11,12] (exceeding 30% on the outer surface).

A minor difference between the modulus of elasticity on the inner and outer surfaces will be required with a thinner shell, but the effect of creating artificial inhomogeneity will be more negligible.

**Figure 4:**
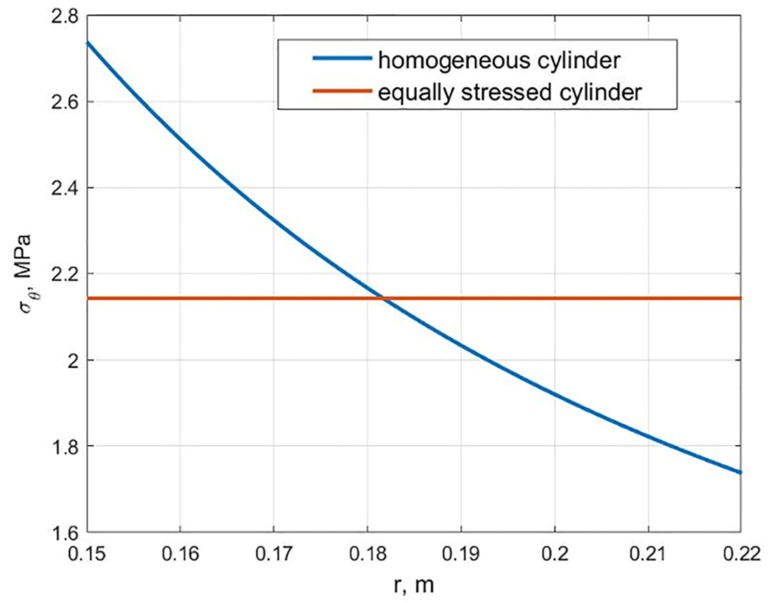
Distribution of stresses *σ_θ_* along the radius for a homogeneous and equally stressed cylinder.

As a result of creating an artificial inhomogeneity, there is a noticeable decrease in the maximum stresses. Figure 4 shows the graphs of the distribution of hoop stresses *σ_θ_* along the radius for a homogeneous cylinder and equally stressed according to the maximum stress failure criterion one. The maximum stresses decreased from 2.73 to 2.14 MPa, i.e., 1.28 times.

The change of the stress–strain state during creep in a cylinder that initially has an equal stress state is discussed below.

**Figure 5:**
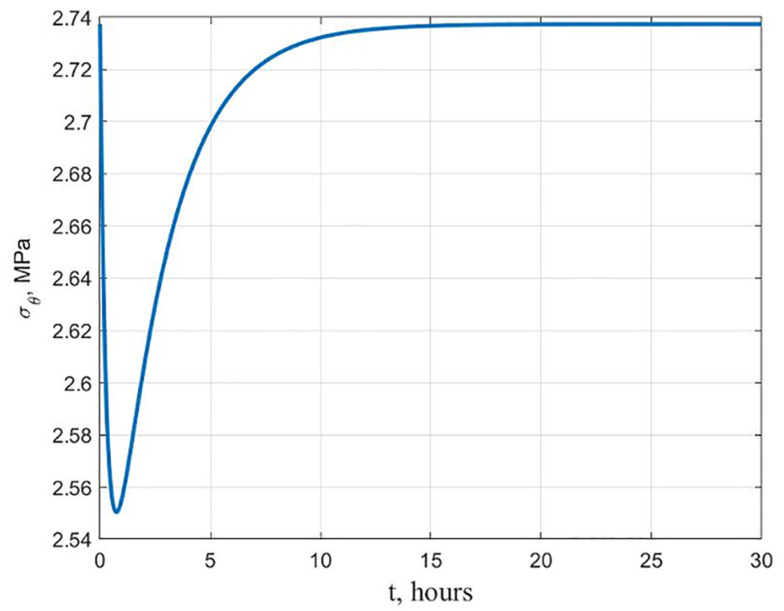
Variation in time of stresses *σ_θ_* at the inner surface of the homogeneous cylinder.

In a homogeneous cylinder, under the action of only a static load during creep, the stresses *σ_θ_* first relax, and then return to the elastic solution (Figure 5). There is the following explanation for this. In [41], it is shown that to obtain a solution at the end of the creep process using the one-term version of the Maxwell–Gurevich equation, the instantaneous constants *E* and *ν* can be replaced in the elastic solution with long-term ones determined by the formulas:

(8) $$\tilde{E}=\frac{E\cdot E_{\infty }}{E+E_{\infty }};\tilde{\nu }=\nu \frac{1+E/\left(2\nu E_{\infty }\right)}{1+E/E_{\infty }}$$

Since the stress distribution in the solution of the Lamé problem does not depend on the elastic constants, at the end of the creep process, it will be the same as at the beginning.

**Figure 6:**
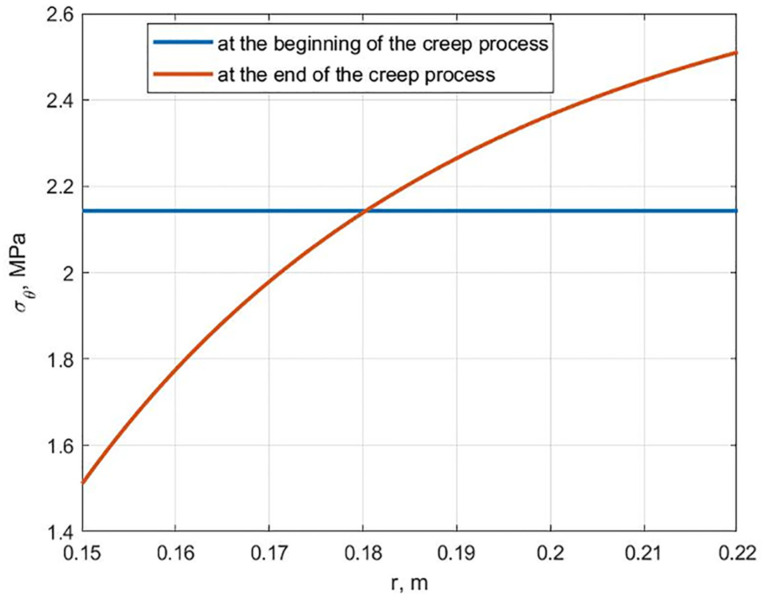
Dependancies of stresses *σ_θ_* along the radius at the beginning and at the end of the creep process for a cylinder equally stressed at the initial moment of time.

**Figure 7:**
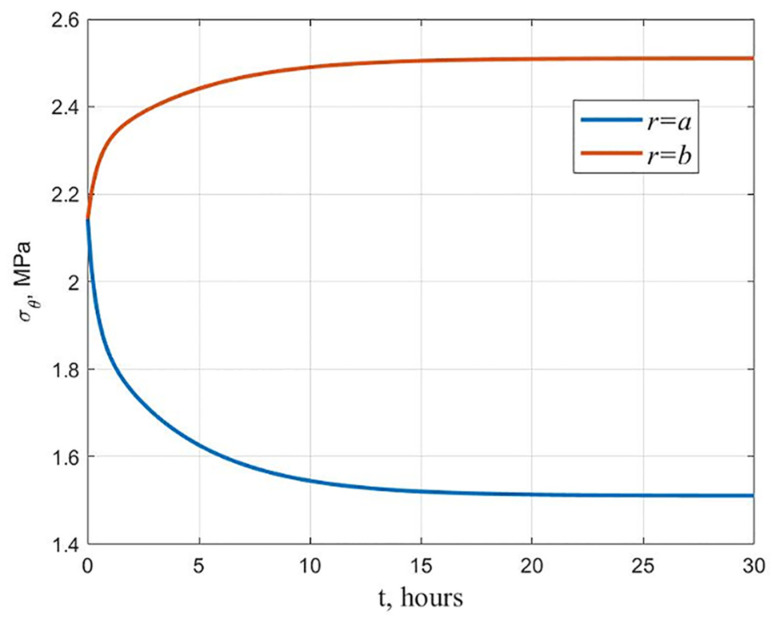
Change in time of stresses *σ_θ_* at the inner and outer surfaces of a cylinder equally stressed at the initial moment of time.

As a result of the cylinder calculation, the hydroxyapatite content changes following Figure 3 (maximum stress criterion). It was found that a cylinder with equal stress at the initial moment ceases to be equally stressed during creep. The graphs of the stresses *σ_θ_* distribution along the radius at the beginning and at the end of the creep process are shown in Figure 6. At the inner surface, the stresses decrease over time, and at the outer surface, they increase, as shown in Figure 7. This is explained by the fact that the modulus of elasticity and the modulus of high elasticity are differently dependent on hydroxyapatite content.

### 3.2. Optimization of the Cylinder Considering Creep

The optimization problem can be set as follows: it is required to find a distribution of the additive content in the structure thickness to be equally stressed at the end of the creep process. The optimization algorithm is similar to the one outlined above, but there are some differences. Instead of the values *E* and ν, it should be operated with long-term constants $\tilde{E}$ and $\tilde{\nu }$. At the first stage, a homogeneous structure is calculated with $\tilde{E}$ = const, $\tilde{\nu }$ = const. Further, the long-term modulus is adjusted according to the formula in (6). The corrected values of $\tilde{E}$ are used to determine the required hydroxyapatite content. Based on the formulas given earlier and (8):

(9) $$\tilde{E}=\frac{E\cdot E_{\infty }}{E+E_{\infty }}=\frac{\left(694+1251\cdot HA\right)\left(228.9+1093\cdot HA\right)}{922.9+2344\cdot HA}$$

With a known value of $\tilde{E}$, this formula represents a quadratic equation relative to the value of *HA*, from which it is easy to find the content of hydroxyapatite.

Then, using the known values of *E* and $E_{\infty }$, the long-term Poisson’s ratio at each node is determined by the second formula in (8).

Thus, at the second and subsequent optimization steps, the long-term modulus of elasticity and the long-term Poisson’s ratio can be considered as a variable along the radius. To determine the stress–strain state the Equation (3) can be used, but the formula should calculate the functions *φ*(*r*) and *ψ*(*r*):

(10) $$\varphi \left(r\right)=\frac{3}{r}-\frac{E^{\prime }}{E}-\frac{2\nu \nu^{\prime }}{1-\nu^{2}};\;\psi \left(r\right)=-\frac{1}{r}\left[\frac{1-2\nu }{1-\nu }\frac{E^{\prime }}{E}+\frac{\left(1+4\nu \right)\nu^{\prime }}{1-\nu^{2}}\right]$$

The finite element method can also be used to calculate the stress–strain state of an inhomogeneous cylinder.

**Figure 8:**
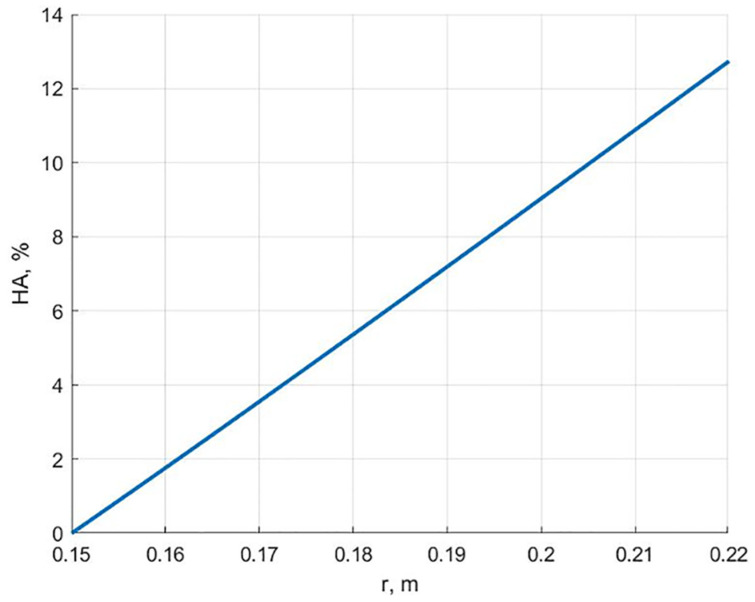
Dependence on the content of hydroxyapatite along the radius for an equally stressed cylinder at the end of the creep process according to the maximum stress failure criterion.

Figure 8 shows the dependence of the hydroxyapatite content along the radius for a cylinder equally stressed according to the maximum stress failure criterion at the end of the creep process. It can be seen from this graph that, in contrast to Figure 3, the maximum additive content is significantly lower.

**Figure 9:**
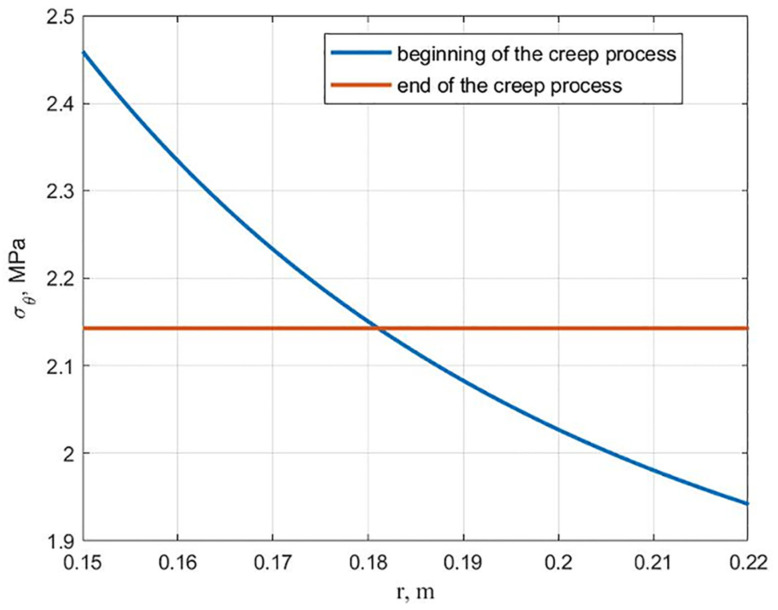
Distribution of stresses *σ_θ_* along the radius for equally stressed cylinder at the end of the creep process.

**Figure 10:**
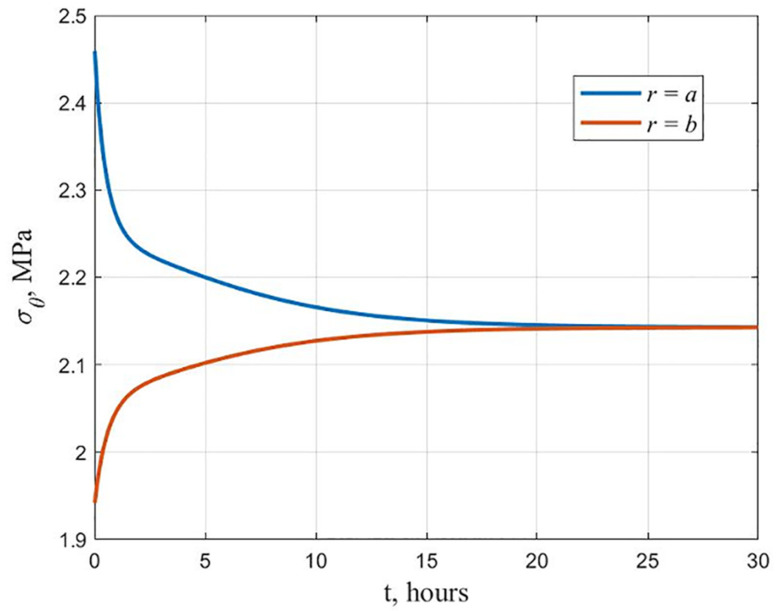
Variation in time of stresses *σ_θ_* for an equally stressed cylinder at the end of the creep process.

The distribution of stresses *σ_θ_* along the radius at the beginning and at the end of the creep process is shown in Figure 9. Figure 10 shows graphs of the hoop stresses at the variation of the inner and outer surface in time. It can be seen from these graphs that at the initial moment, the stresses at the inner surface are higher than at the outer, and in the process of creep at *r* = *a* the stresses decrease, at *r* = *b* they increase, which corresponds to an equal stress state.

**Figure 11:**
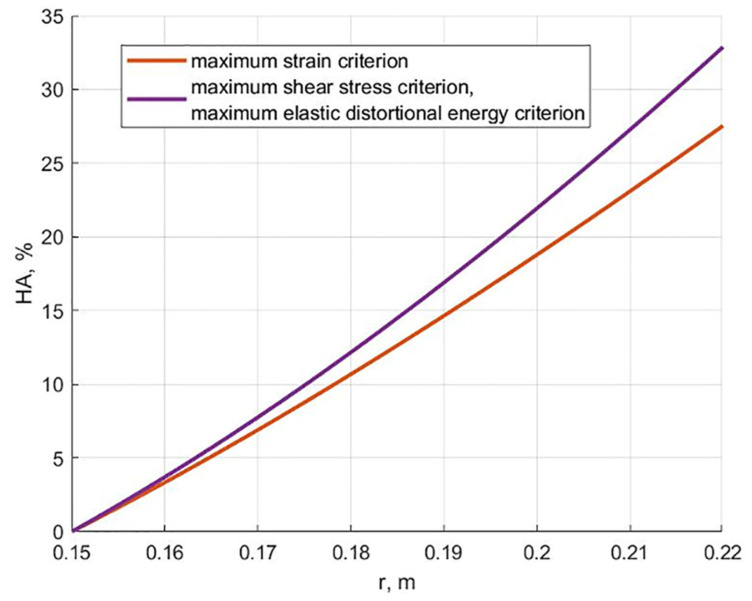
Content of hydroxyapatite depending on the radius for equally stressed cylinders at the end of the creep process according to various cylinder theories.

Figure 11 shows the dependencies in the content of hydroxyapatite for cylinders equally stressed at the end of the creep process, using the criterion of maximum deformation, maximum shear stress, and maximum elastic energy.

## 4. Discussion

Figure 11 shows that cylinders equally stressed at the end of the creep process according to all the considered failure criteria can be created practically without exceeding 30% hydroxyapatite content. The difference between the results based on maximum shear stress and maximum elastic distortional energy failure criteria is insignificant. This can be explained by the fact that the long-term Poisson’s ratio is close to 0.5, and at ν = 0.5, the indicated theories lead to the same result in the case of plane strain.

It should be noted that the proposed models of equally stressed structures, in general, are not of equal strength since the strength of the resulting composite changes with the additives. The algorithm developed in this article, after a minor refinement, allows us to model structures of equal strength. However, it is necessary to know how the strength depends on the content of the additive.

Additionally, the proposed technique allows taking into account the discreteness of the spectrum of polymer relaxation time. This requires experimental data on the dependence of the rheological parameters of the material on the content of additives for two or more members of the spectrum.

For further research, it is of practical interest to construct models of equal strength and equally stressed reinforced concrete structures, taking into account the material’s rheological properties.

## 5. Conclusions

The iterative algorithm is proposed for constructing models of equally stressed polymer cylinders with a finely dispersed mineral filler, taking into account the material’s rheological properties. The optimization problem is theoretically solved by varying the content of the additive along the radius on the basis of four classical failure criteria: the criterion of the maximum stresses, the criterion of the maximum deformations, the criterion of the maximum shear stresses, and the maximum elastic distortional energy (von Mises) criterion. It was found that a cylinder with equal stress in the elastic stage ceases to be uniformly stressed during creep. Furthermore, it is shown that the maximum shear stresses and von Mises criteria lead to practically identical results. The creation of artificial heterogeneity can noticeably decrease the maximum stresses in the thickness of the structure.

## Data Availability

Not applicable.
